# E2F1 and epigenetic modifiers orchestrate breast cancer progression by regulating oxygen-dependent ESRP1 expression

**DOI:** 10.1038/s41389-021-00347-6

**Published:** 2021-08-06

**Authors:** Cheemala Ashok, Neha Ahuja, Subhashis Natua, Jharna Mishra, Atul Samaiya, Sanjeev Shukla

**Affiliations:** 1 Department of Biological Sciences, Indian Institute of Science Education and Research Bhopal, Bhopal, Madhya Pradesh India; 2 Department of Pathology, Bansal Hospital, Bhopal, Madhya Pradesh India; 3 Department of Surgical Oncology, Bansal Hospital, Bhopal, Madhya Pradesh India

**Keywords:** Epigenetics, Breast cancer, Oncogenes

## Abstract

Epithelial splicing regulatory protein 1 (ESRP1) is an RNA binding protein that governs the alternative splicing events related to epithelial phenotypes. ESRP1 contributes significantly at different stages of cancer progression. ESRP1 expression is substantially elevated in carcinoma in situ compared to the normal epithelium, whereas it is drastically ablated in cancer cells within hypoxic niches, which promotes epithelial to mesenchymal transition (EMT). Although a considerable body of research sought to understand the EMT-associated ESRP1 downregulation, the regulatory mechanisms underlying ESRP1 upregulation in primary tumors remained largely uncharted. This study seeks to unveil the regulatory mechanisms that spatiotemporally fine-tune the ESRP1 expression during breast carcinogenesis. Our results reveal that an elevated expression of transcription factor E2F1 and increased CpG hydroxymethylation of the E2F1 binding motif conjointly induce ESRP1 expression in breast carcinoma. However, E2F1 fails to upregulate ESRP1 despite its abundance in oxygen-deprived breast cancer cells. Mechanistically, impelled by the hypoxia-driven reduction of tet methylcytosine dioxygenase 3 (TET3) activity, CpG sites across the E2F1 binding motif lose the hydroxymethylation marks while gaining the de novo methyltransferase-elicited methylation marks. These two oxygen-sensitive epigenetic events work in concert to repel E2F1 from the ESRP1 promoter, thereby diminishing ESRP1 expression under hypoxia. Furthermore, E2F1 skews the cancer spliceome by upregulating splicing factor SRSF7 in hypoxic breast cancer cells. Our findings provide previously unreported mechanistic insights into the plastic nature of ESRP1 expression and insinuate important implications in therapeutics targeting breast cancer progression.

## Introduction

Despite the enormous progress made in the realm of screening, diagnosis, and therapeutic strategies engaged in cancer management, breast cancer remains a major health concern and currently represents a top biomedical research priority. The complex process of breast cancer initiation and progression is associated with a dysregulation in many gene regulatory networks at the transcriptional, post-transcriptional, and epigenetic levels. Interestingly, RNA-binding proteins (RBPs), which govern the post-transcriptional events, including alternative pre-mRNA splicing, polyadenylation, mRNA stability, mRNA localization, and translation, are emerging as critical regulators of several processes in breast carcinogenesis [1–3]. For instance, the RBPs SRSF3, SRSF4, SRSF6, SRSF9, and TRA2β augment mammary cell proliferation and invasion by favoring the splice variants associated with various cancer hallmarks [4–6]. The heterogeneous nuclear ribonucleoprotein M (hnRNPM) promotes epithelial-mesenchymal transition (EMT) and metastasis in breast cancer by promoting the biased expression of *CD44* standard isoform (CD44s) [7]. Similarly, hnRNP A2 increases breast cancer cell invasion by promoting the expression of a specific isoform of *TP53INP2* [8]. Also, RNA binding protein FOX2 (also known as RBM9) drives mesenchymal-specific splicing to regulate EMT [9, 10].

Remarkably, an increasing number of studies has unveiled a central role of Epithelial Splicing Regulatory Protein 1 (ESRP1) in fine-tuning RNA metabolism at different stages of cancer progression. For instance, in colorectal cancer, an elevated level of ESRP1 promotes cancer progression by actuating fibroblast growth factor signaling [11, 12]. ESRP1 is also upregulated in primary ovarian cancer than normal ovarian tissues and promotes cell proliferation and colonization, which is associated with poor patient outcome [13, 14]. Similarly, the higher ESRP1 expression in prostate cancer poses an increased risk of disease progression with an unfavorable prognosis [15]. By analyzing TCGA databases, we also observed a similar overexpression of ESRP1 in breast carcinoma as against normal breast tissues. On the other hand, in a previous study, we have reported that ESRP1 expression is drastically ablated in the hypoxic regions of breast cancer tissues. Consequently, the ESRP1 regulated alternative splicing events go awry to generate aberrant protein isoforms that promote EMT and invasion [16]. Moreover, the association of reduced ESRP1 level with EMT acquisition and invasion is reported in multiple cancer types, including breast cancer [9, 17–24]. Therefore, it is evident that cancer cells leverage the plastic nature of ESRP1 expression incongruously based on oxygen availability in the microenvironment, giving rise to phenotypic and functional heterogeneity.

Thus, the study of molecular mechanisms underlying the oxygen-dependent fine-tuning of ESRP1 expression offers a provocative but as of yet scarcely investigated facet of breast carcinogenesis. In the present study, using both loss- and gain-of-function approaches, we reason that the elevated expression of ESRP1 during breast carcinogenesis and its pro-proliferative activity is effectuated by a concomitant upregulation in transcription factor E2F1 expression. We also demonstrate that ESRP1 is paradoxically downregulated in the hypoxic tumor milieu despite the abundance of E2F1. This phenomenon is explained by our finding that the decreased hydroxymethylation of CpG motifs at the E2F1 binding site abrogates the recruitment of E2F1 on the ESRP1 promoter under hypoxia. In concert with the reduced CpG hydroxymethylation, DNA hypermethylation at the E2F1 binding motif exacerbates ESRP1 diminishment under hypoxia. Furthermore, the functional relevance of E2F1 upregulation in oxygen-deprived breast cancer cells is explicated by the finding that E2F1 alters the cancer spliceome by upregulating splicing factor SRSF7 under hypoxia. Collectively, our study dissects previously unreported mechanistic insights into the expressional plasticity of ESRP1 during breast cancer progression and alludes to important therapeutic interventions.

## Results

### Transcription factor E2F1 is essential for ESRP1 mediated breast carcinogenesis

**Fig. 1:**
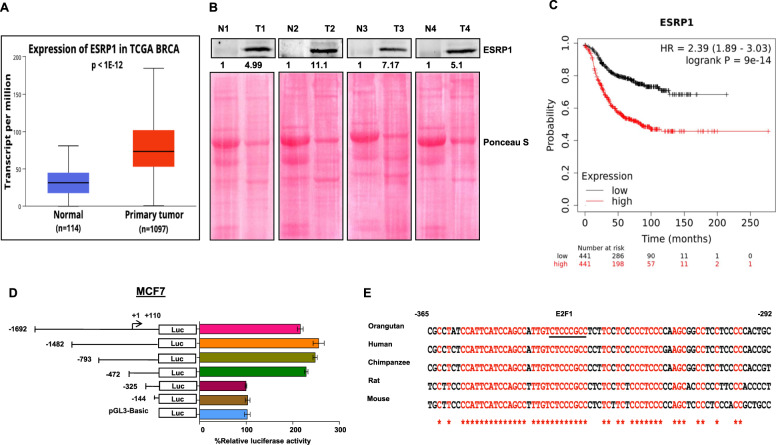
ESRP1 is upregulated in primary breast tumors and is associated with a poor prognosis. **A** TCGA gene expression profile of ESRP1 pertaining to normal breast tissue and primary breast tumor obtained from the UALCAN platform (*P* = 1E–12). and **B** immunoblot of ESRP1 in normal versus breast cancer tissue (*n* = 4). Furthermore, refer to Supplementary Fig. S1B for more samples. **C** Kaplan–Meier Plot for relapse free survival of breast cancer patient comparing the upper (red) and lower (black) quartile ESRP1 expression (Affy ID 225846_at) obtained from www.kmplot.com (Logrank *P* = 9E–14), Hazard ratio = 2.39 (1.89–3.03). **D** Schematic representation of human *ESRP1* promoter analysis in MCF-7 cells. Numbers indicate the position of primers. +1 indicates transcription start site. Deletion constructs of different *ESRP1* promoters and their luciferase activities are shown. **E** Nucleotide sequence alignment of the proximal promoters of orangutan, human, chimpanzee, rat, and mouse ESRP1gene. Putative E2F1 binding site is underlined. Numbers indicate the position of nucleotide sequence corresponding to the human ESRP1 promoter.

A series of recent studies have established an elevated ESRP1 level as a key determinant of tumorigenesis in several cancers, such as colorectal cancer, ovarian cancer, and head and neck cancer. However, ESRP1’s expression pattern and modus operandi during breast carcinogenesis have remained elusive. To examine the mRNA and protein expression pattern of ESRP1 in breast cancer, we analyzed The Cancer Genome Atlas (TCGA) and the Clinical Proteomic Tumor Analysis Consortium (CPTAC) data using the UALCAN platform [25, 26]. Both TCGA and CPTAC data analysis revealed that *ESRP1* gene expression is significantly higher in primary breast tumors than in normal breast tissues (Fig. 1A and Supplementary Fig. S1A). Congruent with this observation, the immunoblot analyses exhibited a consistent upregulation of ESRP1 in breast tumors as compared to paired normal tissues (*n* = 8, *P* = 0.0002) (Fig. 1B and Supplementary Fig. S1B, C). Furthermore, the Kaplan–Meier survival analysis using the TCGA breast invasive carcinoma database revealed that a higher expression of ESRP1 is associated with unfavorable patient outcomes (Fig. 1C). These results prognosticate that ESRP1 is likely to act as an oncogenic driver of breast carcinogenesis. Therefore, it becomes compelling to unveil the regulatory mechanisms that underlie the increased ESRP1 level in the tumor tissues. To identify critical cis-acting elements involved in transcriptional control of ESRP1 expression, we performed promoter deletion analysis using a dual-luciferase reporter system. Reduced luciferase activities from promoter serial deletion fragments suggested vital regulatory elements at positions −472 to −325 bp of the transcription start site (TSS) (Fig. 1D and Supplementary Fig. S2A). Furthermore, this region was scanned for potential transcriptional factor binding using JASPAR [27], which predicted the highly conserved binding site of transcription factor E2F1 with the most decisive relative score (14.5898) (Fig. 1E).

**Fig. 2:**
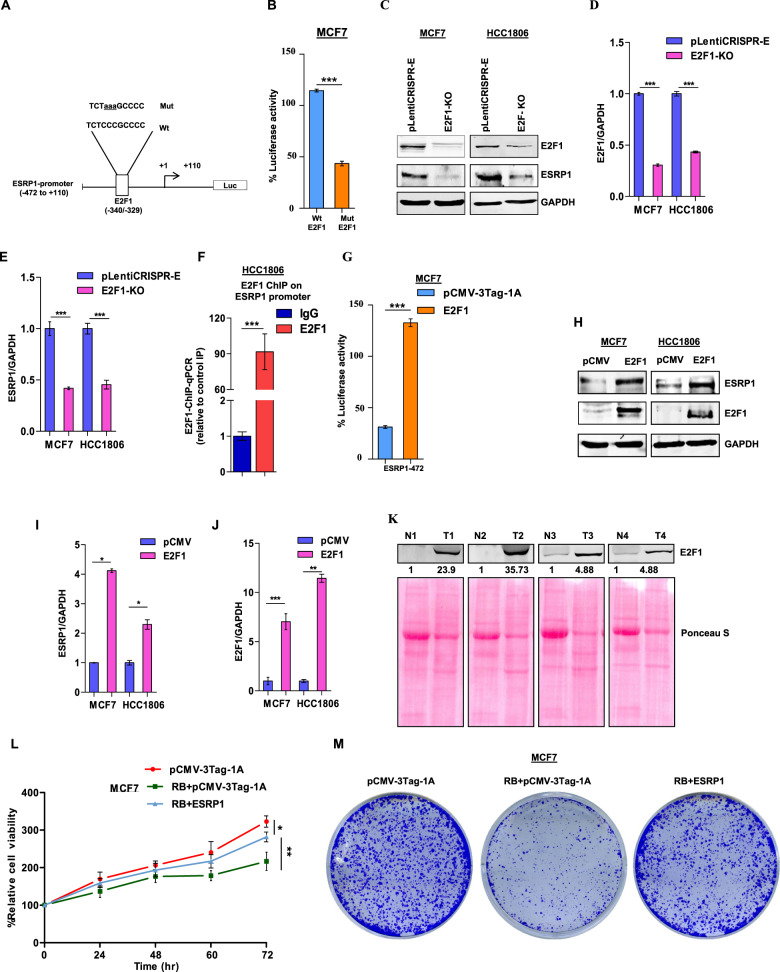
Transcription factor E2F1 is indispensable for ESRP1-mediated breast carcinogenesis. **A** Schematic representation of the ESRP1-472/+110 luciferase reporter construct. The E2F1 (−472*/*+110 bp) sequences for both wild-type and mutated constructs are shown; mutated nucleotides are represented by lowercase letters and underlined. **B** Wild-type or mutant E2F1 luciferase reporter constructs were co-transfected with the Renilla luciferase vector in MCF7 cells, and the luciferase activity was measured after 24 h of transfection. The relative luciferase values are shown as mean ± SD. **C** Immunoblots for E2F1 and ESRP1 in E2F1 knockout MCF7 and HCC1806 cells. **D**, **E** Densitometric analysis of representative blots. **F** ChIP qRT-PCR on ESRP1 promoter using E2F1 antibody in HCC1806 cells. Fold enrichment (E2F1/IgG) was normalized to 5% input. **G** MCF7 cells were co-transfected with ESRP1 (−472*/*+110 bp) promoter construct along with pCMV-3Tag-1A-E2F1 plasmid or pCMV-3Tag-1A as a control. The luciferase activities were measured and the luciferase values are shown. **H** Immunoblots for E2F1 and ESRP1 in E2F1 overexpression cells (MCF7 and HCC1806). **I**, **J** Densitometric analysis of representative blots. **K** Immunoblot of E2F1 in normal versus breast cancer tissue (*n* = 4). **L** Relative cell proliferation was analyzed through MTT assay (*n* = 3) in MCF7 cells. **M** Colony-formation assay of MCF7 cells transfected with the indicated expression vectors were seeded on 6-well plates and after 2 weeks, the colonies were stained with crystal violet. Error bars show mean values ± SD (*n* = 3 unless otherwise specified) calculated using two-tailed Student’s *t* test, *P < 0.05, **P < 0.01 and ***P < 0.001.

To experimentally validate the requirement of E2F1 as a transcriptional activator for ESRP1, we created luciferase reporter constructs of ESRP1 promoter segment −472*/*+110 bp, wherein the E2F1 binding site (TCTCCCGCCCC) is disrupted by site-directed mutagenesis (Fig. 2A). When transfected to MCF7 and HCC1806 cells, the mutated construct exhibited substantially diminished luciferase activity compared to its wild-type counterpart (Fig. 2B and Supplementary Fig. S2B). Furthermore, CRISPR-Cas9 mediated knockout of E2F1 resulted in a severe reduction in ESRP1 expression in both MCF7 and HCC1806 cells (Fig. 2C–E). Moreover, the ChIP assay performed with the anti-E2F1 antibody revealed a remarkable enrichment of E2F1 at the *ESRP1* promoter (Fig. 2F).

Next, to examine the effect of E2F1 overexpression on ESRP1, we ectopically expressed E2F1 in cells harboring ESRP1-472/+110 bp luciferase reporter construct, which led to enhanced luciferase activity (Fig. 2G and Supplementary Fig. S2C). Moreover, enforced E2F1 expression also increased the endogenous ESRP1 expression in breast cancer cell lines (Fig. 2H–J).

Furthermore, to investigate whether E2F1 follows a similar expression pattern in breast cancer as ESRP1, we analyzed TCGA database and observed that E2F1 is significantly upregulated in primary tumors compared to normal tissues (Supplementary Fig. S2D). This observation is also validated experimentally by immunoblotting in breast cancer patient samples (Fig. 2K). Consistent with these findings, correlation analysis of gene expression in TCGA and The Genotype-Tissue Expression (GTEx) data revealed a strong positive correlation between ESRP1 and E2F1 expression in breast cancer (*p* < 0.01, Pearson’s R = 0.54) (Supplementary Fig. S2E). In addition, the Kaplan–Meier survival analysis using the TCGA database revealed that breast cancer patients expressing high levels of E2F1 exhibit a poor prognosis, which is in harmony with ESRP1 (Supplementary Fig. S2F).

This remarkable E2F1-dependency of ESRP1 expression prompted us to investigate whether ESRP1 mediated breast tumorigenesis is also E2F1-dependent. We performed MTT assay and clonogenic assay in MCF7 and HCC1806 cells after ectopically expressing Retinoblastoma (Rb), which restricts E2F1’s access to target promoters. E2F1 sequestration by Rb led to reduced cell viability and proliferation, while these phenotypes are reverted upon ESRP1 rescue in ESRP1 overexpressing cells (Fig. 2L, M and Supplementary Fig. S2G–K). Collectively, these results demonstrate that ESRP1 mediated carcinogenesis is dependent on E2F1-mediated transcriptional activation.

### E2F1 fails to bind the ESRP1 promoter in hypoxic breast cancer cells due to increased DNA methylation

**Fig. 3:**
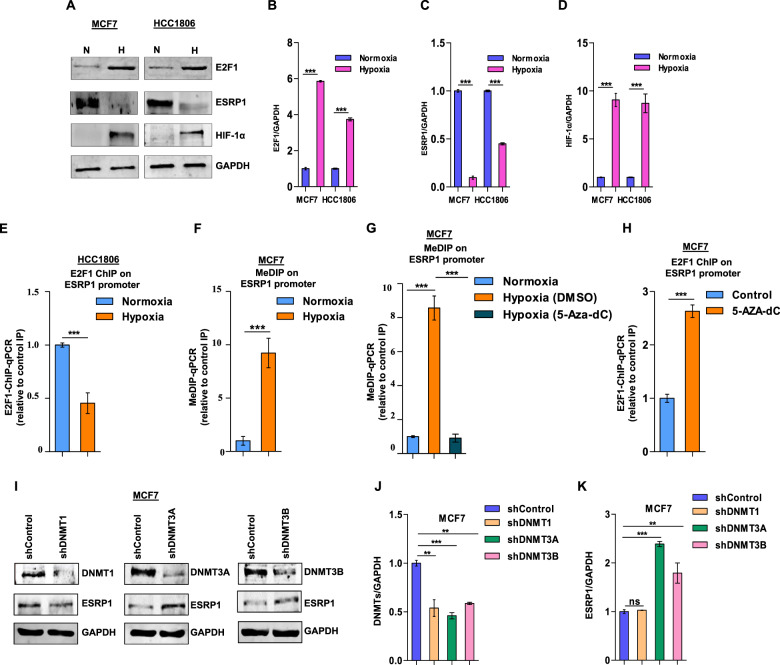
Hypermethylated binding motif repels E2F1 from the ESRP1 promoter in hypoxic breast cancer increased DNA methylation. **A** Immunoblots of E2F1, ESRP1, and HIF1α in MCF7 and HCC1806 cells (Normoxia versus Hypoxia). **B**–**D** Densitometric analysis of representative blots. **E** ChIP qRT-PCR on ESRP1 promoter using E2F1 antibody in HCC1806 cells (Normoxia versus Hypoxia). **F** MeDIP of DNA isolated from MCF7 cells (Normoxia versus Hypoxia) using 5-methyl-cytosine antibody followed by qRT-PCR, relative to input and control IgG (*n* = 3). **G** MeDIP on ESRP1 promoter using 5-methyl-cytosine antibody after treating MCF7 cells with 5-aza-2’-deoxycytidine under hypoxia followed by qRT-PCR, relative to input and control IgG (*n* = 3). **H** ChIP qRT-PCR on ESRP1promoter using E2F1 antibody in 5-aza-2’-deoxycytidine (10 μM) treated MCF7 cells under hypoxia. **I** Immunoblots of DNMT1, DNMT3A, DNMT3B, and ESRP1 protein expression in shDNMT1, shDNMT3A, shDNMT3B, and shcontrol MCF7 cells under hypoxic condition. **J**, **K** Densitometric analysis of representative blots compared to shControl normalized to one. Error bars show mean values ± SD (*n* = 3 unless otherwise specified) calculated using two-tailed Student’s *t* test, **P < 0.01 and ***P < 0.001.

Like any other solid tumor, breast cancer develops regions of reduced tissue oxygen levels due to inadequate vascularization and poor blood circulation [28]. Cancer cells within hypoxic tumor regions often instigate EMT, invasion, and metastasis [29]. Interestingly, we have previously reported that ESRP1 expression is steeply declined by EMT-transcription factors under tumor hypoxia, which, in turn, skews the cellular spliceome to support EMT and invasion [16]. Therefore, we were keen to explore whether the ESRP1 downregulation is accompanied by a concomitant diminishment of E2F1 expression under hypoxia. Paradoxically, however, we observed that E2F1 was further induced under low-oxygen tension (Fig. 3A–D). This discrepancy was explained by our ChIP assay results, which revealed that the E2F1 recruitment on the ESRP1 promoter is compromised under hypoxia (Fig. 3E). Given that hypoxia plays a critical role in DNA hypermethylation [30], we suspected that the reduced E2F1 recruitment on the *ESRP1* promoter could be a consequence of the increased methylation of the E2F1 binding site. As expected, our MedIP results indicate that CpG islands around the E2F1 binding site on the *ESRP1* promoter are heavily methylated under hypoxia as against normoxia (Fig. 3F). In addition, breast cancer cells treated with 5-aza-2′-deoxycytidine (5-Aza-dc), an established DNA methylation inhibitor [31], reduced the methylation level of the E2F1 binding site (Fig. 3G), which, in turn, restored the E2F1 binding on the ESRP1 promoter (Fig. 3H).

Next, we sought to identify the DNA methyltransferases (DNMTs) responsible for hypoxia-driven hypermethylation of the E2F1 binding site. The shRNA-mediated knockdown of DNMT3A and 3B, but not DNMT1, resulted in increased expression of ESRP1 under hypoxia (Fig. 3I–K, and Supplementary Fig. S3A–E). Interestingly, despite a slight reduction in the DNMT3A/B expression under hypoxia (Supplementary Fig. S3F, G), 5-mC levels at the E2F1 binding site were significantly elevated (Fig. 3F).

Therefore, these results suggest that de novo methyltransferases are critical in restricting E2F1 from inducing ESRP1 expression in the hypoxic breast cancer cells.

### Decreased hydroxymethylation of the E2F1 binding site contributes to ESRP1 downregulation under hypoxia

**Fig. 4:**
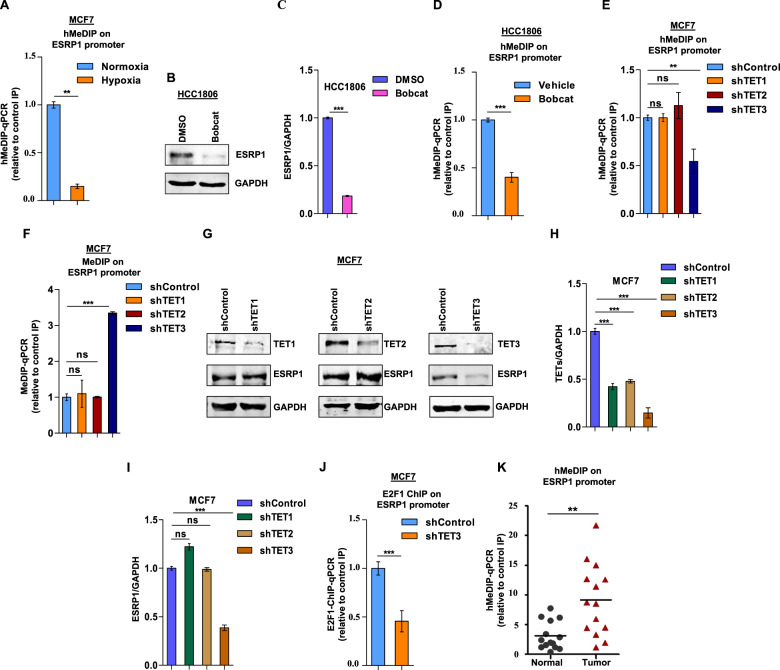
Reduced CpG hydroxymethylation at the E2F1 binding motif contributes to ESRP1 downregulation under hypoxia. **A** hMeDIP of DNA isolated from MCF7 cells (Normoxia versus Hypoxia) using 5 hydroxymethylcytosine antibody followed by qRT-PCR, relative to input and control IgG (*n* = 3). **B** Immunoblots of ESRP1 after bobcat (70–90 μM) treatment under normoxia in HCC1806. **C** Densitometric analysis of representative blots. **D** hMeDIP in HCC1806 after bobcat (70–90 μM) treatment under normoxia, followed by qRT-PCR relative to input and control IgG (*n* = 3). **E** hMeDIP in MCF7 cells transfected with shRNA against TET1, TET2, TET3 versus shcontrol cells under normoxia, followed by qRT-PCR relative to input and control IgG (*n* = 3). **F** MeDIP in MCF7 cells transfected with shRNA against TET1, TET2, TET3 versus shcontrol cells under normoxia, followed by qRT-PCR relative to input and control IgG (*n* = 3). **G** Immunoblots of TET1, TET2, TET3, and ESRP1 protein expression in shTET1, shTET2, shTET3, and shcontrol MCF7 cells under normoxic condition. **H**, **I** Densitometric analysis of representative blots compared to shControl normalized to one. **J** ChIP qRT-PCR on ESRP1 promoter using E2F1 antibody in TET3 knockdown MCF7 cells under normoxia. Fold enrichment (E2F1/IgG) was normalized to 5% input. **K** hMeDIP in normal and breast tumor tissue genomic DNA and qRT-PCR of ESRP1promoter region relative to input and control IgG (*n* = 14). Error bars show mean values ± SD (*n* = 3 unless otherwise specified) calculated using two-tailed Student’s *t* test, ns (non-significant), **P < 0.01 and ***P < 0.001.

Like DNA methylation, DNA hydroxymethylation is also highly influenced by tumor hypoxia and plays an essential role in hypoxia-specific gene expression [30]. Therefore, we sought to understand any involvement of altered 5hmC dynamics in regulating E2F1 recruitment under hypoxia. The hydroxymethylated DNA immunoprecipitation (hmedIP) experiments performed with MCF7 cells revealed that the 5hmC levels are drastically reduced under hypoxia at the E2F1 binding site on the ESRP1 promoter (Fig. 4A). The oxygen-dependent ten-eleven translocation (TET) proteins are the key enzymes that catalyze DNA demethylation through 5-methylcytosine oxidation. Hence, to understand the underlying mechanism of decreased 5hmC level, we explored the TET enzymes for their involvement in E2F1-mediated ESRP1 expression. When a general TET inhibitor called Bobcat [32] was dosed onto HCC1806 and MCF7 cells, the ESRP1 expression was severely ablated (Fig. 4B, C and Supplementary Fig. S4A, B) as a result of decreased 5hmC (Fig. 4D and Supplementary Fig. S4C), and a concomitant increase in 5mC level (Supplementary Fig. S4D). Furthermore, we delved into screening all three TET enzymes (TET1, TET2, and TET3) for their specificity towards the E2F1-ESRP1 axis. When all the TETs are knocked down individually in normoxic cells, only TET3-knockdown resulted in decreased 5hmC and elevated 5mC level at the E2F1 binding site on the ESRP1 promoter (Fig. 4E, F and Supplementary Fig. S4E, F). Congruently, only TET3 knockdown led to reduced ESRP1 expression (Fig. 4G–I and Supplementary Fig. S4G–I), as a result of reduced E2F1 recruitment on the ESRP1 promoter (Fig. 4J). Moreover, TET3 knocked-down cells under normoxia exhibited an identical alternative splicing pattern of ESRP1 targets (*hMENA, SLK, SCRIB, RALGPS2, SLC37A2, FNIP1, CD44*, and *ARHGEF1*) as the hypoxic breast cancer cells (Supplementary Fig. S4J), (Supplementary Table S1). However, it is worth noting that the hypoxia-induced changes in the TET3 expression are not consistent between MCF7 and HCC1806 cell (Supplementary Fig. S4K, L). These data collectively portray that TET3-mediated hydroxymethylation of CpG sites across the E2F1 binding motif is indispensable for E2F1-mediated ESRP1 upregulation during carcinogenesis, and a diminished TET3 activity contributes to the declined ESRP1 expression under hypoxia. This is further seconded by our hMedIP assay results exhibiting a greater 5hmC level at E2F1 binding motif in breast tumor samples as against normal tissues (Fig. 4K).

### E2F1 skews cancer spliceome by upregulating splicing factor SRSF7 in hypoxic breast cancer cells

**Fig. 5:**
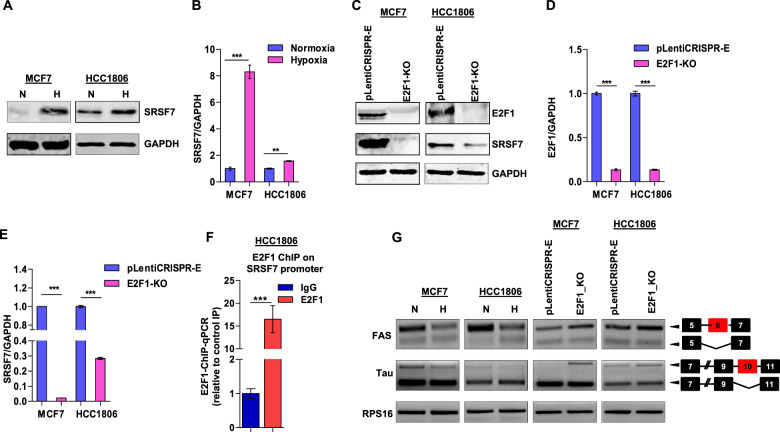
E2F1 alters the cancer spliceome by inducing splicing factor SRSF7 expression in hypoxic breast cancer cells. **A** Immunoblots of SRSF7 in MCF7 and HCC1806 cells (Normoxia versus Hypoxia). **B** Densitometric analysis of representative blots. **C** Immunoblots for E2F1 and SRSF7 in E2F1 knockout MCF7 and HCC1806 cells under hypoxia. **D**, **E** Densitometric analysis of representative blots. **F** ChIP qRT-PCR on SRSF7 promoter using E2F1 antibody in HCC1806 cells under hypoxia. Fold enrichment (E2F1/IgG) was normalized to 5% input. **G** Semi-quantitative PCR of FAS and Tau genes after 48 h of hypoxic treatment and E2F1 knockout under hypoxia in MCF7 and HCC1806 cells (RPS16 used as a control). **P < 0.01 and ***P < 0.001.

After dissecting the molecular basis of inefficient E2F1 binding on the ESRP1 promoter under hypoxia, we sought to elucidate the significance of elevated E2F1 expression in oxygen-deprived cells. We hypothesized that E2F1 might transcriptionally induce the RBPs other than ESRP1 to support hypoxic adjustments to the cancer spliceome [33]. To address this, first, we identified the E2F1-targeted RBPs after analyzing the published E2F1 ChIP-seq data (GSM935477). Next, we screened these RBPs for their differential expression under hypoxia versus normoxia by immunoblot analysis (Supplementary Fig. S5A, B). Among all the RBPs (HNRNPU, HNRNPK, RBM5, HNRNPLL, HNRNPH1, HNRNPM, HNRNPA2B1) screened, only SRSF7 (also known as 9G8) was substantially upregulated in hypoxia-treated breast cancer cells (Fig. 5A, B). To experimentally validate the E2F1-dependence of SRSF7 expression, we performed CRISPR/Cas9-mediated knockout of E2F1, which resulted in a severe reduction of SRSF7 (Fig. 5C–E). Furthermore, our ChIP assay results confirmed the binding of E2F1 on the SRSF7 promoter (Fig. 5F). Next, we investigated the role of E2F1 in orchestrating the alternative splicing of SRSF7-targeted pre-mRNAs such as *Fas* [34] and *Tau* [35, 36] under hypoxia. E2F1 knockout resulted in the reversal of the hypoxia-specific alternative splicing scheme by increasing the inclusion of *Fas* exon 6 and *Tau* exon 10, both of which would otherwise be excluded by SRSF7 in hypoxic cells (Fig. 5G). Notably, the biased expression of shorter Fas isoform (*FasΔEx6*) under the influence of the hypoxia-driven E2F1-SRSF7 axis renders the hypoxic cancer cells uniquely suited for bypassing programmed cell death. Our in vivo data also confirms increased expression of SRSF7 in hypoxic tumor regions (Supplementary Fig. S5C). Therefore, it is evident from these results that E2F1 plays an important role in skewing the cancer spliceome to meet the altered adaptive requirement in the hypoxic tumor niches.

## Discussion

The aberrant expression of RBPs in many human cancers with diverse underlying mechanisms contributes to accelerated tumor progression by regulating RNA metabolism at several levels, including alternative splicing, thus emphasizing the need for finely-tuned expression of RBPs in the cell. ESRP1, a member of the RBM family of RBPs, was initially identified as epithelium-specific splicing regulators in a genome-wide high-throughput cDNA screen aimed at finding the key regulators of *FGFR2* splicing in epithelial cells [22]. ESRP1 mediate the alternative splicing of a number of genes associated with actin dynamics, cell polarity, and cell-cell adhesion during EMT [37]. Recently, a panoply of research has established the elevated ESRP1 level as a significant determinant of tumorigenesis in several cancers, such as colorectal cancer, ovarian cancer, and head and neck cancer. In line with these observations, our present work also found that ESRP1 is weakly expressed in normal breast epithelium, whereas its expression level in carcinoma in situ was substantially elevated. Interestingly, in a previous report, we have demonstrated that ESRP1 expression is shackled in the hypoxic regions of breast tumor, and consequently, hypoxic epithelial cells attain mesenchymal phenotype due to a dramatic re-organization of actin dynamics. In addition, a large body of research has reported the diminishment of ESRP1 level during EMT acquisition and invasion as a result of upregulation in EMT-associated transcription factors, such as δEF1, SIP1, Snail, Slug, and Twist [21, 22, 38, 39].

Thus, the expression pattern of ESRP1 at different stages of cancer progression is phenomenally plastic, and this plasticity seems to play a critical role in the spatiotemporal optimization of cancer spliceome. However, despite substantial efforts to understand the regulatory mechanism of ESRP1 downregulation during EMT, the factors involved in the up-regulation of ESRP1 during tumorigenesis remained to be fully elucidated. In our quest to unveil the trans-acting regulators of ESRP1 upregulation, we analyzed the ESRP1 promoter, and our results demonstrate that the binding of transcription factor E2F1 is essential for ESRP1 expression. Notably, similar to ESRP1, E2F1 also gets upregulated in the primary breast tumor as opposed to normal breast tissues. However, the mechanism of E2F1 upregulation under hypoxia still remains to be determined.

Next, we asked whether a decrease in E2F1 level is observed upon hypoxia treatment following the expression pattern of ESRP1. Unexpectedly, E2F1 expression was further enhanced in hypoxic cells than in normoxic cells. To reason this discrepancy, we investigated the possible alteration in binding efficiency of E2F1 and found that the E2F1 recruitment on the ESRP1 promoter is compromised under hypoxia despite its abundant expression. This phenomenon is further explained by our finding that the E2F1 binding is sensitive to DNA methylation and hydroxymethylation. More specifically, oxygen deprivation coerces the E2F1 binding site to lose hydroxymethyl marks owing to diminished TET3 activity, which follows a concomitant gaining of DNMT3A/3B-mediated methyl marks. Furthermore, hypoxia-induced changes in TET3 levels are inconsistent among different cell lines while maintaining a consistently low 5hmC level at the E2F1 motif on ESRP1 promoter under hypoxia. These results commensurate with a previous report that demonstrated that reduction in oxygen availability lowers the 5hmC level due to impaired TET activity, independently of TET expression [30]. Of note, the expression of DNMT3A/3B is only modestly affected under hypoxia, while the increase in 5mC levels at the E2F1 motif is significant. These observations strengthen our hypothesis that the loss of hydroxymethyl marks readily favors the recruitment of de novo methyltransferases, irrespective of their expression levels. Altogether, our study revealed how the epigenetic marks fine-tune the recruitment of E2F1 on ESRP1 promoter, and it is highly likely that the methylation/hydroxymethylation status of the E2F1 motif on the ESRP1 promoter demonstrates a distinct pattern, irrespective of the expressional changes of epigenetic modifiers under hypoxia at a global level.

**Fig. 6:**
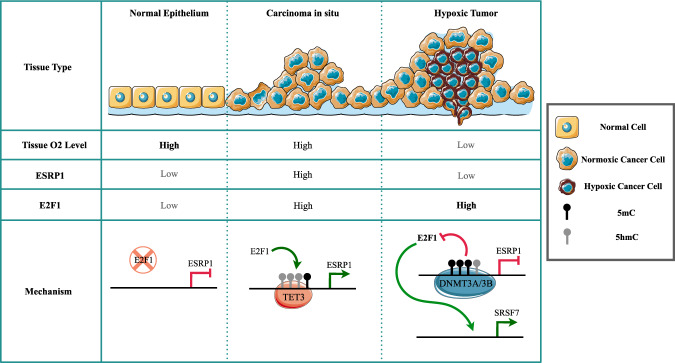
The expression of ESRP1 is substantially elevated in breast carcinoma-in-situ compared to normal breast tissue. The elevated levels of transcriptional activator E2F1 and gain of 5hmC marks on the ESRP1 promoter govern the ESRP1 upregulation. However, ESRP1 expression is severely diminished within the hypoxic tumor niche despite a high E2F1 level. Mechanistically, hypoxia-driven reduction in TET3 activity coerces the E2F1 binding site on the ESRP1 promoter to lose 5hmC marks while gaining DNMT3A/3B-dependent 5mC marks. Additionally, elevated E2F1 regulates the hypoxia-specific spliceome by inducing SRSF7 expression.

In addition, the knockdown of TET3 essentially mimicked tumor hypoxia to effectuate the pro-EMT splicing switch of ESRP1 targets. We further explored the possible role of E2F1 in influencing the transcription of splicing factors other than ESRP1 under hypoxia. It turned out that the elevated E2F1 level under hypoxia is actually essential for the expression of splicing factor SRSF7, which in turn contributes to the spliceomic adaptation in response to tumor hypoxia. Collectively, our findings unveil the regulatory mechanisms underlying the plastic nature of ESRP1 expression during breast carcinogenesis (Fig. 6) and insinuate novel therapeutic interventions.

## Materials and methods

### Cell culture

Human breast cancer cell lines MCF7 and HCC1806 were obtained from American Type Culture Collection (ATCC) and were cultured in ATCC recommended DMEM and RPMI media respectively supplemented with 10% fetal bovine serum (FBS; Sigma, F7524), 100 units/ml of penicillin and streptomycin (Invitrogen, 15140122) and 2 mmol/l L-glutamine (Sigma, G7513). These cell lines were cultured in a humidified atmosphere at 37◦C and 5% CO_2_. For treatment under hypoxic conditions (1% O_2_), cells were kept in a Ruskinn INVIVO2 400 hypoxia chamber. For TET activity inhibition, Bobcat339 (70–90 μM) (Sigma, SIML2611) and DNMTs inhibitor 5-Aza-2′-deoxycytidine (10 μM) (Sigma, A3656) were added in the media.

### Bioinformatics analyses

The Cancer Genome Atlas (TCGA) gene expression profile of ESRP1 and E2F1, and Clinical Proteomic Tumor Analysis Consortium (CPTAC) data for ESRP1 pertaining to normal breast tissue and primary breast tumor were obtained from the university of Alabama cancer (UALCAN) platform (http://ualcan.path.uab.edu) [25, 26]. Prognostic values of ESRP1 (Affymetrix ID 225846_at) and E2F1(Affymetrix ID 204947_at) in breast cancer are analyzed by web platform Kaplan–Meier Plotter (www.kmplot.com). The cohorts are divided into high- and low-expression groups according to upper and lower quartile gene expression and then two groups are compared in terms of relapse free survival [40]. The pairwise correlation analysis between mRNA expression of E2F1 and ESRP1 was performed on GEPIA web tool using TCGA BRCA and GTex database [41].

### RNA interference

The MCF7 and HCC1806 cells (2 × 10^5^) were seeded and after 24 h cells were infected with lentivirus containing small hairpin RNA (shRNA) (Sigma, Mission Human Genome shRNA Library) against Tet1, Tet2, Tet3, DNMT1, DNMT3a, DNMT3b, and shControl with 8 µg/ml polybrene (Sigma, H9268) containing media. Cells were selected using 1 µg/ml puromycin (Sigma, P9620) for 3 days. For rescue experiments, overexpression of Rb1 and ESRP1 was done using Lipofectamine 2000 reagent (Invitrogen, 11668019) as per the manufacturer’s instructions. The list of shRNAs used in this study is given in Supplementary Table S2.

### Generation of gene specific cDNA clones, promoter deletion constructs and site-directed Mutagenesis

ESRP1- pCMV-3Tag-1A clone was generated by PCR-amplified full-length ESRP1 fragment using cDNA derived from MCF7 cells as a template. This PCR-amplified full-length ESRP1 fragment was cloned into pCMV-3Tag1a (Agilent, 240195) vector between BamHI F and HindIII R restriction sites. E2F1 and RB1 expression plasmids were a kind gift by Dr. Sudhakar Baluchamy, Department of Biotechnology, Pondicherry University, INDIA. The details of the primers are listed in Supplementary Table S3.

To generate promoter constructs, human ESRP1 (Gene ID: 54845) promoter sequence was retrieved from the Eukaryotic Promoter Database (EPD) (https://epd.vital-it.ch/). Different lengths of ESRP1 promoter constructs were PCR amplified from genomic DNA derived from MCF7 cells and inserted/ligated into pGL3 basic vector (Promega) between KpnI and NheI restriction sites. The primers used in promoter constructs are listed in Supplementary Table S4.

The site-directed mutant construct of the ESRP1 promoter was prepared using oligonucleotides harboring mutations in the E2F1 binding site. The wild type ESRP1 −472 promoters was used as a template. The ESRP1 promoter SDM was confirmed by DNA sequencing after the digestion of non mutated vector with the endonuclease DpnI (TaKaRa, 1235 A). The details of the oligonucleotides are listed in Supplementary Table S5.

### Luciferase reporter assays

The luciferase reporter assays were performed as described previously [42]. Briefly, MCF7 and HCC1806 cells (0.05 ×10^6^) were seeded in 24-well plates and cultured for 16 h. The cells were co-transfected with different ESRP1 promoter–luciferase constructs along with pRL-TK Renilla luciferase plasmid (Promega, E2231). After 48 h transfection, cells were harvested and lysed and then subjected to detection of luciferase activity using the GloMax- Multi Detection System (Promega), and the values were normalized to Renilla luciferase activities. The relative values are represented as mean ±SD of triplicates from a representative experiment.

### Cell viability assay

The in vitro cell viability test was performed with a MTT assay as described previously [43, 44]. 24 h post transfection, cells were seeded in 96-well culture plates (4 × 10^3^/well) for 12 h, 24 h, 36 h, 48 h, and 60 h (in triplicate for each condition). 20 µl MTT (Sigma, Saint Louis, USA) stock solution (5 mg/ml) was added to each well and incubated for 2–3 h. Then, the supernatant was removed, followed by the solubilisation of MTT crystals using DMSO and the absorbance was measured at 570 nm using plate reader BioTek Eon (BioTek, Winooski, USA).

### Colony formation assay

Colony formation assay was performed using standard protocol [45, 46]. 24 h post transfection, cells were seeded in 6-well culture plates at low density (~1000 cells per well) and cultured for 7 days. The cells were fixed using methanol and acetic acid (3:1) for 5 min at RT followed by staining with 0.1% crystal violet in 10% ethanol. Results were shown as the mean value ± SD error using GraphPad Prism. The assay was repeated three times.

### Chromatin immunoprecipitation (ChIP) assay

Chromatin immunoprecipitation reaction was performed using standard protocol [47]. Briefly, the nuclei were isolated from MCF7 and HCC1806 cells, followed by sonication of the lysates to shear the DNA (approximate average size was 200–500 bp), DNA-protein complexes in the lysates were subjected to immunoprecipitation using anti-E2F1(Abcam, ab179445, lot no. GR155150-29) or normal rabbit IgG (Millipore, 12- 370, lot no. 2295402). Immunoprecipitated protein-DNA complexes were reverse crosslinked, chromatin-bound DNA was purified using PCR purification kit (QIAGEN, 28106) and measured by qRT-PCR using ESRP1 promoter-specific primers (listed in Supplementary Table S6). All the ChIP experiments were performed at least twice. IP values were normalized to input using the following formula: 2^ (Ct_input − Ct_immunoprecipitation). The significance between two different groups was identified using Student’s *t* test. *P* < 0.05 was considered statistically significant.

### Methylated DNA immunoprecipitation (MeDIP) and hydroxymethylated DNA immunoprecipitation (hMeDIP)

Genomic DNA was isolated from HCC1806 and MCF7 cells using genomic DNA isolation kit (Sigma, G1N350) according to manufacturer’s instruction. MeDIP and hMeDIP assays were performed as described previously [47]. Briefly, 3 µg of sonicated DNA was processed and incubated with anti-5-Methyl cytosine (CST, D3S27, lot no. 1) or anti-5-hydoxymethyl cytosine (CST, 51660 S, lot no. 1) antibodies along with normal rabbit IgG or normal mouse IgG (Millipore, 12- 371B, lot no. 2332526) for overnight at 4 °C. Immunoprecipitated fractions and 5% input were analyzed by quantitative real-time PCR in triplicate using the SYBR Green Master Mix (Promega, A6002, lot no. 0000385100) and specific primers (listed in Supplementary Table S6). All the experiments were performed at least thrice. IP values were normalized to input using the following formula: 2^ (Ct_input—Ct_immunoprecipitation). The significance between two different groups was identified using Student’s *t* test. *P* < 0.05 was considered statistically significant.

### sgRNA Target design and cloning

The design of the sgRNAs was done using the online tool GPP sgRNA Designer (https://portals.broadinstitute.org/gpp/public/analysis-tools/sgrna-design). The sgRNAs targeting E2F1 were cloned into the BbsI site of the lentiviral vector pLentiCRISPR-E (Addgene, 78852) as described previously [16]. The details of the oligonucleotides utilized for CRISPR*/*Cas9-mediated knockout are listed in Supplementary Table S7.

### Immunoblotting

The cells were lysed using urea lysis buffer (8 M urea, 2 M thiourea, 2% CHAPS, 1% DTT) and 1× PIC (leupeptin 10–100 M, pepstatin 1 M, 1–10 mM EDTA, <1 mM AEBSF), spun at 14,000 × *g* in a 4 °C centrifuge. The supernatant was separated, quantified and equal concentration of protein samples was loaded. Quantification of the bands was done using ImageJ software. Details of antibodies used for immunoblotting are provided in Supplementary Table S8.

### RNA isolation and Semi-quantitative PCR

RNA isolation was done with TRIzol (Invitrogen, 15596026) according to the manufacturer’s guidelines. 2 μg RNA were used for cDNA synthesis by PrimeScript 1st strand cDNA Synthesis Kit (TaKaRa, 6110 A, lot no. AJX1015N). Semi-quantitative PCR were performed as described previously [16]. The details of the oligonucleotides are listed in Supplementary Table S9.

### E2F1 ChIP-Seq analysis

ChIP-Seq dataset for E2F1 performed using MCF7 cells was downloaded from GEO (Accession Number: GSM935477). Transcription start sites (TSS) for all splicing factors was downloaded from BioMart by Ensembl. In order to look for E2F1 binding site in the promoter region of splicing factors, bedtools toolset was used to assign nearest ChIP-Seq peak to splicing factor TSS. A region of 2000 base pairs upstream or downstream of TSS was considered as the promoter region. The list of splicing factors with E2F1 ChIP-Seq peaks in their promoter region has been provided in Supplementary Table S10.

### Immunohistochemistry

The study was approved by the Institute Ethics Committee of the Indian Institute of Science Education and Research Bhopal, India. Informed consent was obtained from all the patients. Formalin-fixed, paraffin-embedded human breast cancer tissue sections were obtained from Bansal Hospital, Bhopal, India. Immunohistochemistry was performed according to the experimental protocol of the Super Sensitive™* Polymer-HRP Detection System (Catalog no.- QD430-XAKE) and staining was visualized with the DAB (3,3′-diaminobenzidine, Sigma) chromogenic method and counterstained with Harris’ hematoxylin (Merck). Slides were fixed for 2 h at 65°C in the heat bath, deparaffinized, and rehydrated as per the standard procedure. In total, 10 mM sodium citrate buffer (pH 6)-based antigen retrieval was done in the laboratory microwave for 14 min. Endogenous peroxidase was quenched with 1:10 dilution of 3% hydrogen peroxidase in methanol, followed by blocking with 1% bovine serum albumin (BSA). Primary antibodies against CAIX (1:50) and SRSF7 (1:20) were used (details of the antibodies are provided in Supplementary Table S8). Sections were examined using the Thermo Scientific™ Invitrogen™ EVOS™ FL Auto 2 Imaging System and at ×40 magnification. Images were then processed in Adobe Photoshop CS Version 8.0.

### Breast Cancer sample collection

Tumor and adjacent normal tissue pairs were collected from patients undergoing surgery for breast cancer at Bansal Hospital, Bhopal, India. The study was approved by the Institute Ethics Committee of Indian Institute of Science Education and Research Bhopal. Informed consent was obtained from all the patients. The tissue samples were snap frozen immediately after surgery and stored at −80°C until use. Clinical characteristics of patients used in the study are presented in Supplementary Table S11.

### Statistics

All statistical tests were performed with Prism Graph Pad 5 (GraphPad Software, La Jolla, USA). In the bar graph, differences between two groups were compared using an unpaired two-tailed Student’s *t* test. *P *<* 0.05, **P *<* 0.01, and ***P *<* 0.001 indicate statistical significance, ns non-significant difference (*P* > 0.05).

## Supplementary information

Supplemental Material

Supplementary Table S10

## Data Availability

All the data are included in the manuscript.
