# Supplementary material for: E2F1 and epigenetic modifiers orchestrate breast cancer progression by regulating oxygen-dependent ESRP1 expression
Source: Oncogenesis. 2021 Aug 6;10(8):58. doi: 10.1038/s41389-021-00347-6 (PMC8346533; doi:10.1038/s41389-021-00347-6)
Supplement: Supplementary file 1 — Supplemental Material [file 41389_2021_347_MOESM1_ESM.pdf]

E2F1 and epigenetic modifiers orchestrate breast cancer progression by regulating oxygen-dependent ESRP1 expression

Cheemala Ashok[1,†],Neha Ahuja[1,†], Subhashis Natua[1], Jharna Mishra[2], Atul Samaiya[3] and Sanjeev Shukla [1,*]

Supplementary Information

Supplementary Table S1. Alternative splicing events of ESRP1 target genes in MCF7 and HCC1806 cells. (Normoxia versus Hypoxia) and (shcontrol versus shTET3) [1].

**Gene symbol**	**Type of ASE in Warzecha et al [1] in low ESRP1**	**Normal vs Hypoxia MCF7**	**shcontrol vs shTET3 MCF7**	**Normal vs Hypoxia HCC1806**	**shcontrol vs shTET3 HCC1806**
hMENA	Skip	Skip	Skip	Skip	Skip
SLK	Skip	Skip	Skip	Skip	Skip
SCRIB	Inc	Skip	Skip	Skip	Inc
RALGPS2	Skip	Skip	Skip	Skip	Skip
SLC37A2	Skip	Skip	Skip	Skip	Skip
FNIP1	Skip	Skip	Skip	Skip	Skip
CD44	NC	Skip	Skip	Skip	Skip
ARHGEF11	Inc	Inc	Inc	Inc	Inc

Supplementary Table S2. Oligo sequence of shRNAs.

shControl	CCGGCGCTGAGTACTTCGAAATGTCCTCGAGGACATTTCGAAGTACTCAGCGTTTTT
shDNMT1	CCGGCGACTACATCAAAGGCAGCAACTCGAGTTGCTGCCTTTGATGTAGTCGTTTTT
shDMNT3A	CCGGCCACCAGAAGAAGAGAAGAATCTCGAGATTCTTCTCTTCTTCTGGTGGTTTTTG

shDNMT3B	CCGGCCATGCAACGATCTCTCAAATCTCGAGATTTGAGAGATCGTTGCATGGTTTTTG
shTET1	CCGGCCTATATGTATGGCACAATATCTCGAGATATTGTGCCATACATATAGGTTTTTG
shTET2	CCGGCCTCAAGCATAACCCACCAATCTCGAGATTGGTGGGTTATGCTTGAGGTTTTTTG
shTET3	CCGGGAACCTTCTCTTGCGCTATTTCTCGAGAAATAGCGCAAGAGAAGGTTCTTTTTG

Supplementary Table S3. List of primers used in ESRP1 cloning.

S.NO	Primers	Sequence
1	ESRP1- BamHIF	CGGGATCCATGACGGCCTCTCCGGATTA
2	ESRP1- HindIII R	CCCAAGCTTTTAAATACAAACCCATTCTTTGGG

Supplementary Table S4. List of primers used in ESRP1 promoter cloning.

S.NO	Primers	Sequence
1	ESRP1-1692 Fw	GGGGTACCCGCCTCCGCCTGCACCTTCT
2	ESRP1- 1482 Fw	GGGGTACCGGCTGGACACCTAGAGCCGA
3	ESRP1- 793 Fw	GGGGTACCGGCTCGCAGGATTTCTCCTG
4	ESRP1- 472 Fw	GGGGTACCGAGCCCTTTACCTCTCTGAGC
5	ESRP1- 325 Fw	GGGGTACCCTCCCCCTCCCGAAGCGGCC
6	ESRP1-144 Fw	GGGGTACCGCAGCCTTGCTCCAGGCTT
7	ESRP1+110 Rev	CGGCTAGCAGGCGGTAAGGTGGTGTGGA

Supplementary Table S5. List of primers used in site directed mutagenesis (SDM).

S.NO	Primers	Sequence
1	E2F1 SDM Fw	CCAGCCATTGTCTAAATCCCCTTCCTCCCCCT
2	ESF1 SDM Rev	AGGGGGAGGAAGGGGATTTAGACAATGGCTGG

Supplementary Table S6. List of primers used in Chromatin immunoprecipitation (ChIP), MeDIP and hMeDIP.

S.NO	Primers	Sequence
1	ESRP1 promoter Fw	GAGCCCTTTACCTCTCTGAGC
2	ESRP1 promoter Rev	TTCAAACCACGACGTGGCAGC
3	SRSF7 promoter Fw	GAGCTGGAGTCTTGGGCGAG
4	SRSF7 promoter Rev	ACCCATGAGTCCCGGCAG

Supplementary Table S7. List of Oligo sequences utilized for CRISPR/Cas9-mediated knockout.

S.NO	Primers	Sequence
1	sgRNA E2F1 Fw	CACCGGGAGATGATGACGATCTGCG
2	sgRNA E2F1 Rev	AAACCGCAGATCGTCATCATCTCCC

Supplementary Table S8. List of antibodies utilized for Immunohistochemistry, and Immunoblotting.

S.No	Antibody	Company	Catalog no	Lot no
1	GAPDH (D16h11)	CST	5174S	7
2	RBM35A (ESRP1)	Abcam	ab107278	GR279719-9
3	E2F1	Abcam<br>Sigma	ab179445<br>05-379	GR155150-29<br>3280452
4	DNMT1	Abcam	ab87656	GR215476-4
5	DNMT3A	Abcam	ab2805	GR218985-4
6	DNMT3B	R&D systems	MAB7646	CHLT0115041
7	TET1	Novus Biologicals	NBP215135	41386

8	TET2	CST	45010	1
9	TET3	Novus Biologicals	NBP220602	41185
10	HIF-1α (D2U3T)	CST	14179S	3
11	SRSF7	Abcam	ab170679	GR179666-4
12	HNRPA2/B1	Abcam	ab6102	GR125277-56
13	Normal Rabbit IgG	CST	2729S	8
14	Normal Mouse IgG	CST	5415S	10
15	5-Methylcytosine (5-mC)	CST	D3S27	1
16	5-Hydroxymethylcytosine (5-hmC)	CST	51660S	1
17	Alexa-Flour 680 anti-rabbit IgG	Invitrogen	A32734	RJ243414
18	Alexa-Flour 800 anti-mouse IgG	Invitrogen	A32730	SC243837
19	HNRNP LL	CST	4783S	2
20	HNRNP U	abcam	ab172608	YK030623CS
21	HNRNP K	CST	9081S	1
22	HNRNP M1-M4	abcam	ab177957	GR141541-3
23	HNRNP H	abcam	ab10374	GR250291-24
24	RBM5	abcam	ab85504	GR16494-7
25	CAIX	abcam	ab184006	GR173128-25

Supplementary Table S9. List of primer sequences utilized for Semi-quantitative PCR.

**S.NO**	**Primers**	**Sequence**
1	RPS16 Fw	AAACGCGGCAATGGTCTCATCAAG
2	RPS16 Rev	TGGAGATGGACTGACGGATAGCAT
3	hMENA Fw	GAATTGCTGAAAAGGGATC
4	hMENA Rev	CTGTTCCTCTATGCAGTATTTGAC
5	SLK Fw	TTGAGCAGGAAATGATGAGGAAAA

6	SLK Rev	CTGCCTTCTGCTGCTGGATGA
7	SCRIB Fw	GACAAGGAGGGGGCCGTGGTTTCT
8	SCRIB Rev	TATGCCCTCGTCGTCCCCCTTAT
9	RALGPS2 Fw	AGACCTCATGGCCTGCTTTTGAAA
10	RALGPS2 Rev	TGTAGGCTTTTTGCCTTCTTTTAA
11	SLC37A2 Fw	CTGGAAGGTGTCCCTGAGCA
12	SLC37A2 Rev	TGAACAAGCAAGAGTCTGAGCA
13	FNIP1 Fw	AACACAGTTATTAATGGACTGCTTGG
14	FNIP1 Rev	GTGCTATGCCACTGTCTCTGTC
15	CD44 Fw	CTCCACCTGAAGAAGATTGTACATC
16	CD44 Rev	TCAGATCCATGAGTGGTATGGGACC
17	ARHGEF11 Fw	GGCAGCAGGAGGTTACAAAGTT
18	ARHGEF11 Rev	TGAGTGGTCGGTGCTTGAGTC
19	FAS Fw	CACCAAGTGCAAAGAGGAAG
20	FAS Rev	GGAGATTCATGAGAACCTTGG
21	Tau Fw	CAACGCCACCAGGATTCCAGCAAA
22	Tau Rev	ATGTTGCCTAATGAGCCACACTTG

Supplementary Table S11. Clinical characteristics of patients

S.No	Patient No.	Histopathology	Estrogen (ER), Progesterone (PR), Her2 Status
1	Patient 1	Carcinoma ypT4N0Mx	ER(-ve) PR(-ve) Her2(weak+ve)
2	Patient 2	Infiltrating duct carcinoma pT2N0Mx	ER(weak+ve) PR(weak+ve) Her2(-ve)
3	Patient 3	Infiltrating duct carcinoma Grade II pT2N1Mx	ER(-ve) PR(-ve) Her2(+ve)
4	Patient 4	Infiltrating duct carcinoma Grade II pT3N2Mx	ER(+ve) PR(+ve) Her2(-ve)

5	Patient 5	Infiltrating duct carcinoma Grade I pT2N0Mx	ER(+ve) PR(+ve) Her2(-ve)
6	Patient 6	Infiltrating duct carcinoma NOS type grade II Left Breast	ER(weak+ve) PR(+ve) Her2(+ve)
7	Patient 7	Infiltrating duct carcinoma GradeII pT2N0Mx	ER(+ve) PR(+ve) Her2(-ve)
8	Patient 8	Infiltrating duct Carcinoma pT2N0Mx	ER(+ve) PR(weak+ve) Her2(-ve)
9	Patient 9	Grade III invasive Duct Carcinoma	-
10	Patient 10	Invasive Duct Carcinoma	-
11	Patient 11	Infiltrating Duct Carcinoma	-
12	Patient 12	Infiltrating Duct Carcinoma Grade II pT3N0Mx	-
13	Patient 13	Infiltrating duct carcinoma grade III Pathological stage pT3N0Mx	ER(-ve) PR(-ve) Her2(-ve)
14	Patient 14	Mucinous carcinoma pT2SnN0Mx	ER(+ve) PR(+ve) Her2(-ve)
15	Patient 15	Mixed metaplastic carcinoma pathological stage pT3N0Mx	ER(Weak +ve) PR(moderate +ve) Her2(-ve)
16	Patient 16	Invasive carcinoma, grade II, pT2N0Mx	ER(+ve) PR(+ve) Her2(equivocal)
17	Patient 17	Invasive carcinoma, grade II, pT2N0Mx	ER(-ve) PR(-ve) Her2(-ve)
18	Patient 18	Invasive carcinoma, grade II, pT2N1Mx	ER(+ve) PR(+ve) Her2(-ve)
19	Patient 19	Invasive carcinoma, grade III, pT2N2Mx	ER(-ve) PR(-ve) Her2(+ve)

Reference

1 Warzecha CC *et al* (2010). An ESRP‐regulated splicing programme is abrogated during the epithelial‐mesenchymal transition. *The EMBO journal* **29:** 3286-3300.

Supplementary Figures

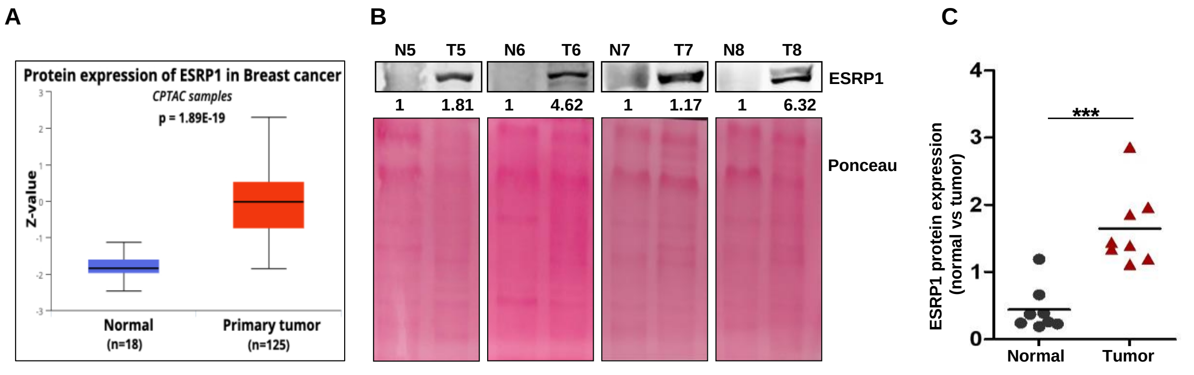

Supplementary Figure S1. ESRP1 is upregulated in primary breast tumors and is associated with a poor prognosis. (A) The Clinical Proteomic Tumor Analysis Consortium (CPTAC) data for ESRP1 pertaining to normal breast tissue and primary breast tumor, obtained from the UALCAN platform. **(B)** Immunoblot of ESRP1 in normal versus breast cancer tissue. **(C)** Quantification for ESRP1 protein expression for breast tumor versus normal tissues of 8 breast cancer patients (n = 8, *P* =0.0002).

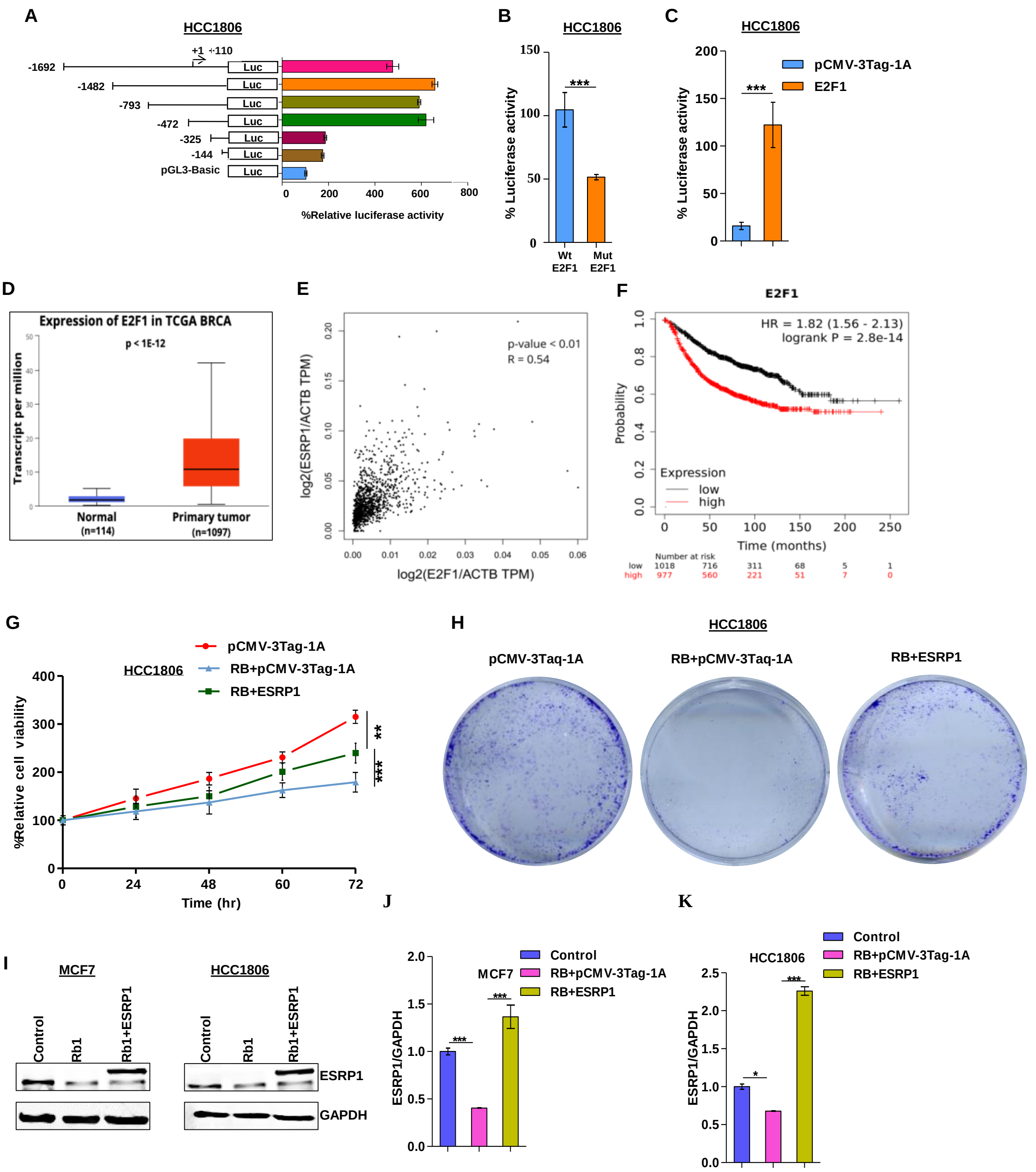

Supplementary Figure S2. Transcription factor E2F1 is indispensable for ESRP1-mediated breast carcinogenesis. (A) Schematic representation of human *ESRP1* promoter analysis in HCC1806 cells. Numbers indicate the position of primers. +1 indicates transcription start site. Deletion constructs of different *ESRP1* promoters and their luciferase activities are shown. **(B)** Wild-type or mutant E2F1 luciferase reporter constructs were co-transfected with the Renilla luciferase vector in HCC1806 cells, and the luciferase activity was measured after 24 h of transfection. The luciferase values are shown as mean ± SD. **(C)** HCC1806 cells were co-transfected with ESRP1 (−472/+110 bp) promoter construct

along with pCMV-3Tag-1A-E2F1 plasmid or pCMV-3Tag-1A as a control. The luciferase activities were measured and the relative luciferase values are shown. Error bars show mean values ± SD (n = 3 unless otherwise specified) calculated using two-tailed Student's t-test, ***P < 0.001. **(D)** TCGA gene expression profile of E2F1 pertaining to normal breast tissue and primary breast tumor obtained from the UALCAN platform (P = 1E-12). **(E)** The Pearson's correlation analysis between mRNA expression of E2F1 and ESRP1 normalized to β-Actin from GEPIA web tool using TCGA BRCA and GTex database (P < 0.01, R = 0.54)**. (F)** Kaplan-Meier Plot for relapse free survival of breast cancer patient comparing the upper (red) and lower (black) quartile E2F1 expression (Affy ID: 204947_at) obtained from www.kmplot.com (Logrank P = 2.8E-14 , Hazard ratio = 1.82 (1.56-2.13). **(G)** Relative cell proliferation was analyzed through MTT assay (n = 3) in HCC1806. **(H)** Colony-formation assay of HCC1806 cells transfected with the indicated expression vectors were seeded on 6-well plates and after 2 weeks, the colonies were stained with crystal violet. **(I)** Immunoblot of ESRP1 to confirm overexpression of ESRP1 in MCF7 and HCC1806 cells. **(J and K)** Densitometric analysis of representative blots. Error bars show mean values ± SD (n = 3 unless otherwise specified) calculated using two-tailed Student's t-test, *P < 0.05, **P < 0.01 and ***P < 0.001.

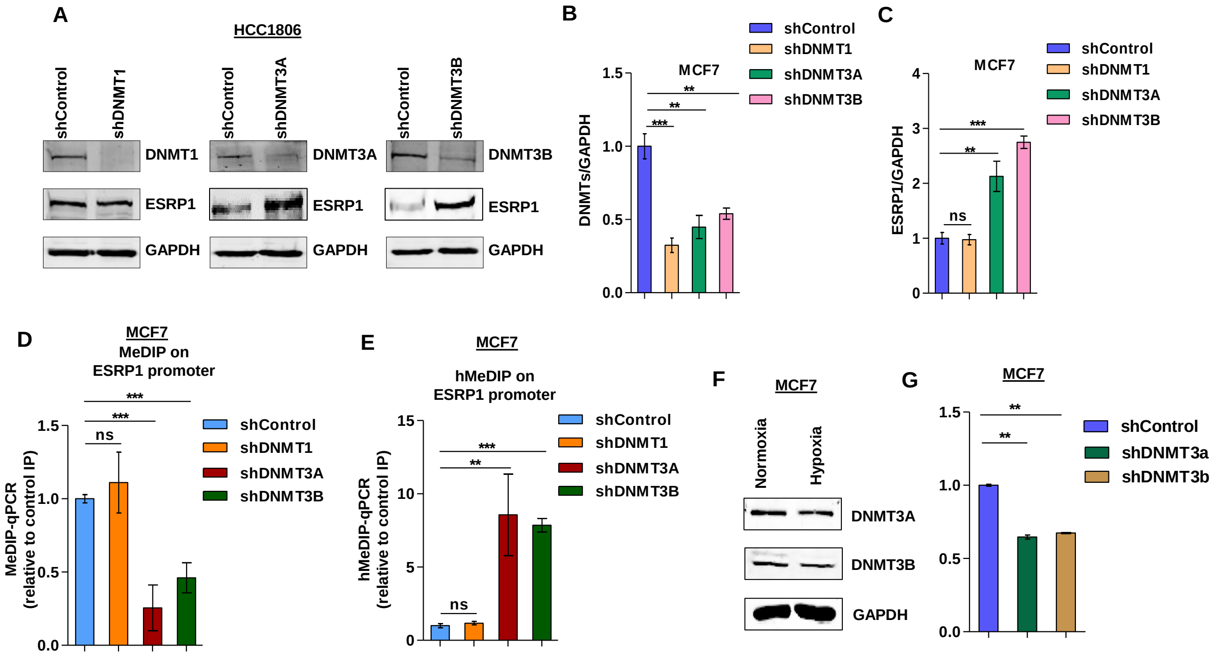

Supplementary Figure S3. Hypermethylated binding motif repels E2F1 from the ESRP1 promoter in hypoxic breast cancer. increased DNA methylation. (A) Immunoblots of DNMT1, DNMT3A, DNMT3B, and ESRP1 protein expression in shDNMT1, shDNMT3A, shDNMT3B, and shcontrol HCC1806 cells under hypoxic condition. **(B and C)** Densitometric analysis of representative blots compared to shControl normalized to one. **(D)** MeDIP in MCF7 cells transfected with shRNA against DNMT1, DNMT3A, DNMT3B versus shcontrol cells under hypoxia, followed by qRT-PCR relative to input and control IgG (n = 3). **(E)** hMeDIP in MCF7 cells transfected with shRNA against DNMT1, DNMT3A, DNMT3B versus shcontrol cells under hypoxia, followed by qRT-PCR relative to input and control IgG (n = 3). **(F)** Immunoblots of DNMT3a and DNMT3b protein expression in MCF7 cells under normoxic vs hypoxic condition. **(G)** Densitometric analysis of representative blots compared to shControl normalized to one. Error bars show mean values ± SD (n = 3 unless otherwise specified) calculated using two-tailed Student's t-test, ns (non significant), $^{**}P < 0.01$ and $^{***}P < 0.001$.

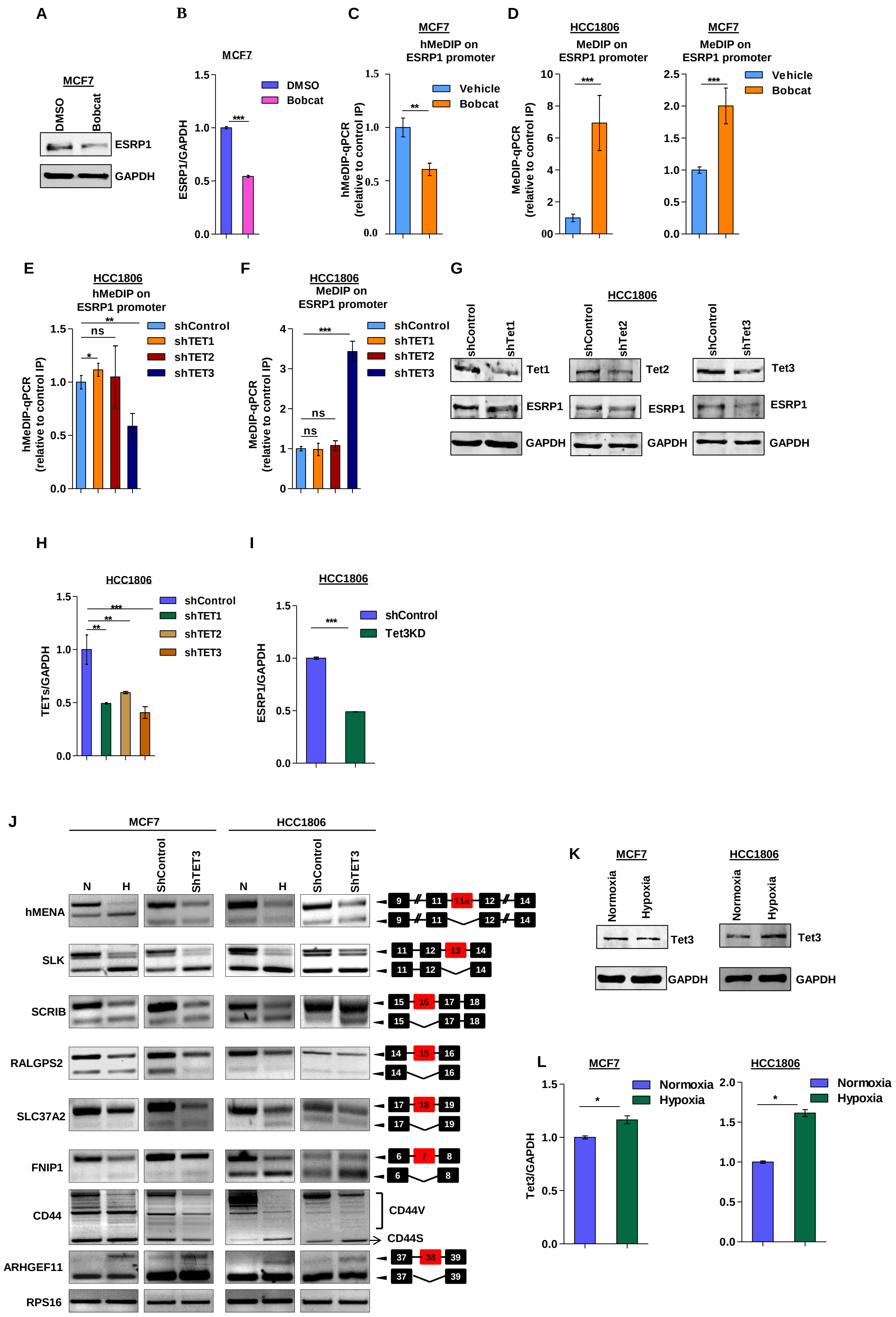
A
MCF7
DMSO
Bobcat
ESRP1
GAPDH
B
MCF7
DMSO
Bobcat
ESRP1/GAPDH
***
C
MCF7
hMeDIP on
ESRP1 promoter
hMeDIP-qPCR
(relative to control IP)
Vehicle
Bobcat
**
D
HCC1806
MeDIP on
ESRP1 promoter
MCF7
MeDIP on
ESRP1 promoter
MeDIP-qPCR
(relative to control IP)
Vehicle
Bobcat
***
E
HCC1806
hMeDIP on
ESRP1 promoter
hMeDIP-qPCR
(relative to control IP)
shControl
shTET1
shTET2
shTET3
ns
*
**
F
HCC1806
MeDIP on
ESRP1 promoter
MeDIP-qPCR
(relative to control IP)
shControl
shTET1
shTET2
shTET3
***
ns
G
HCC1806
shControl
shTet1
shTet2
shTet3
Tet1
Tet2
Tet3
ESRP1
GAPDH
H
HCC1806
TETs/GAPDH
shControl
shTET1
shTET2
shTET3
I
HCC1806
ESRP1/GAPDH
shControl
Tet3KD
J
MCF7
HCC1806
N
H
ShControl
ShTET3
hMENA
SLK
SCRIB
RALGPS2
SLC37A2
FNIP1
CD44
ARHGEF11
RPS16
CD44V
CD44S
K
MCF7
HCC1806
Normoxia
Hypoxia
Tet3
GAPDH
L
MCF7
HCC1806
Tet3/GAPDH
Normoxia
Hypoxia
*

Supplementary Figure S4. Reduced CpG hydroxymethylation at the E2F1 binding motif contributes to ESRP1 downregulation under hypoxia. (A) Immunoblots of ESRP1 after bobcat (70-90 μM) treatment under normoxia in MCF7 cells. **(B)** Densitometric analysis of representative blots. **(C)** hMeDIP in MCF7 cells after bobcat (70-90 μM) treatment under normoxia, followed by qRT-PCR relative to input and control IgG (n = 3). **(D)** MeDIP in HCC1806 and MCF7 cells after bobcat (70-90 μM) treatment under normoxia, followed by qRT-PCR relative to input and control IgG (n = 3). **(E)** hMeDIP in HCC1806 cells transfected with shRNA against TET1, TET2, TET3 versus shcontrol cells under normoxia, followed by qRT-PCR relative to input and control IgG (n = 3). **(F)** MeDIP in HCC1806 cells transfected with shRNA against TET1, TET2, TET3 versus shcontrol cells under normoxia, followed by qRT-PCR relative to input and control IgG (n = 3). **(G)** Immunoblots of TET1, TET2, TET3, and ESRP1 protein expression in shTET1, shTET2, shTET3, and shcontrol HCC1806 cells under normoxic condition. **(H and I)** Densitometric analysis of representative blots compared to shControl normalized to one. **(J)** Semi-quantitative PCR of *hMENA, SLK, SCRIB, RALGPS2, SLC37A2, FNIP1, CD44* and *ARHGEF1* after 48h of hypoxic treatment and TET3 knockdown in MCF7 and HCC1806 cells (RPS16 used as a control). **(K)** Immunoblots of TET3 protein expression in MCF7 and HCC1806 cells under normoxic vs hypoxic condition. **(L)** Densitometric analysis of representative blots compared to shControl normalized to one. Error bars show mean values ± SD (n = 3 unless otherwise specified) calculated using two-tailed Student's t-test, ns (non significant), $^{*}P < 0.05$, $^{**}P < 0.01$ and $^{***}P < 0.001$.

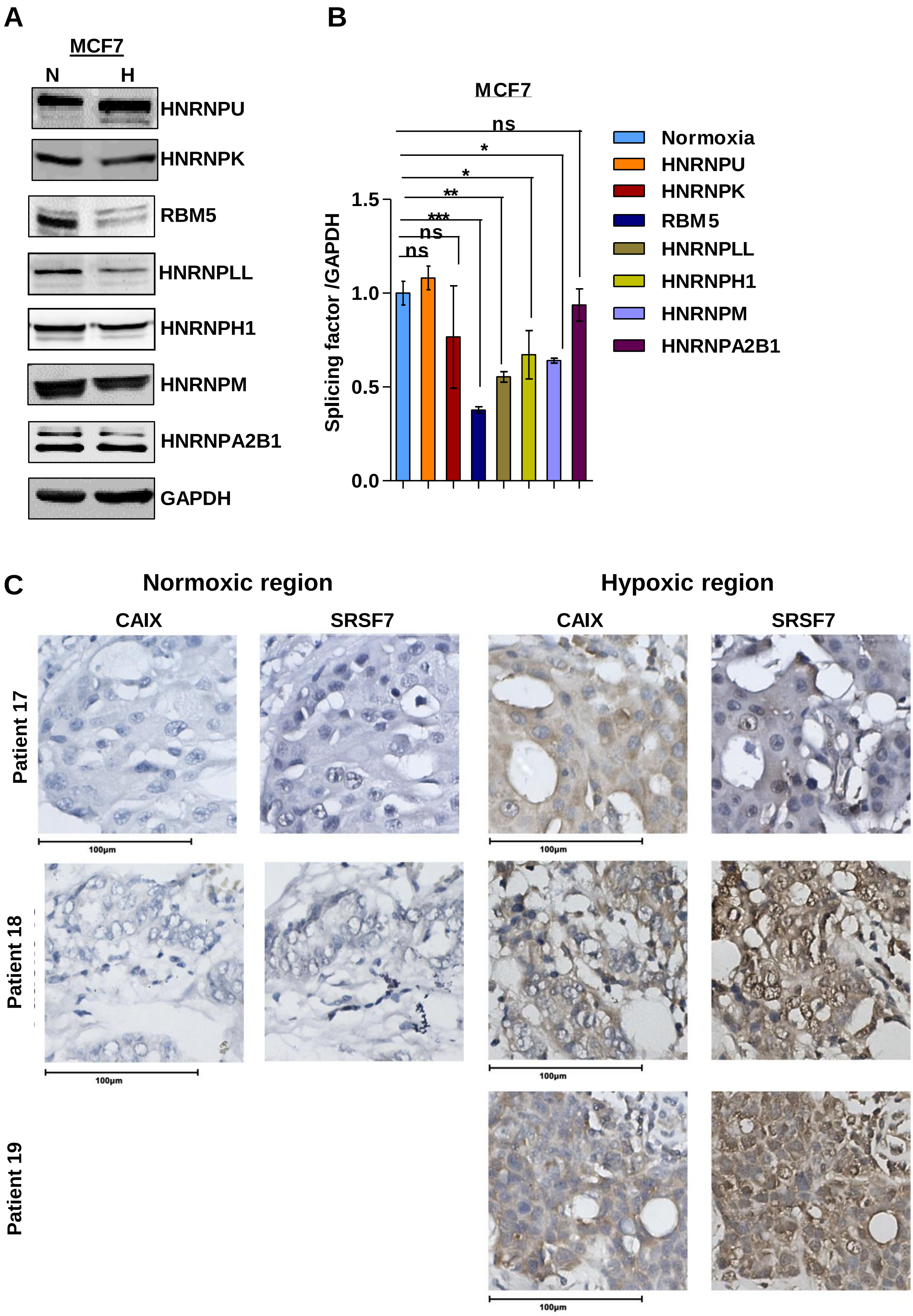

136 **Supplementary Figure S5. E2F1 alters the cancer spliceome by inducing splicing factor SRSF7**

expression in hypoxic breast cancer cells. (A) Immunoblots of HNRNPU, HNRNPK, RBM5, HNRNPLL, HNRNPH1, HNRNPM, HNRNPA2B1 in MCF7 (Normoxia versus Hypoxia). **(B)** Densitometric analysis of representative blots compared to normoxia normalized to one. **(C)** CAIX and SRSF7 immunostaining of three illustrative cases of breast cancer patients. Hypoxic regions: areas representing strong membranous and/or cytoplasmic immunostaining for CAIX also exhibit strong expression of SRSF7. Normoxic regions: areas representing weak/no immunostaining for CAIX also exhibit weak expression of SRSF7. Magnification: 40X. Error bars show mean values ± SD (n = 3 unless otherwise specified) calculated using two-tailed Student's t-test, ns (non significant), *$P < 0.05$, **$P < 0.01$ and ***$P < 0.001$.
